# Internet and game behaviour at a secondary school and a newly developed health promotion programme: a prospective study

**DOI:** 10.1186/1471-2458-10-544

**Published:** 2010-09-09

**Authors:** J Rob J de Leeuw, Marieke de Bruijn, Gerdien H de Weert-van Oene, August JP Schrijvers

**Affiliations:** 1 Julius Center, Division of Public Health, Utrecht University, Utrecht, The Netherlands

## Abstract

**Background:**

This study investigated the Internet and game use of secondary school children, the compulsiveness of their use and the relationship with other health behaviours. It also evaluated the preliminary results of a recently developed school health promotion programme, implemented at a secondary school in the Netherlands in January 2008. This programme is one of the first to combine seven health behaviours in one educational programme and is a pilot project for a case-control study.

**Methods:**

A total of 475 secondary school children completed an extensive questionnaire before and a year after starting the programme. Of these children, 367 were in first, second and third grade; the grades in which the lessons about internet and game behaviour were implemented. Questionnaires contained questions about personal information, Internet and game use (Compulsive Internet Use Scale), and other health behaviours (alcohol use, physical activity, psychosocial wellbeing and body mass index).

**Results:**

Heavy Internet use was significantly associated with psychosocial problems, and heavy game use was significantly associated with psychosocial problems and less physical activity. No relationship was found with alcohol use or body mass index. The time spent on Internet (hours/day) and the number of pathological Internet users increased during the study. The number of game users decreased but heavy game use increased.

**Conclusion:**

The association between heavy Internet use and psychosocial problems and between game use and psychosocial problems and less physical activity emphasizes the need to target different health behaviours in one health education programme. A case-control study is needed to further assess the programme-induced changes in Internet and game behaviour of school children.

## Background

The value of schools as a place for health education is recognized worldwide, and a growing number of studies have demonstrated that school health promotion can lead to positive change, improving the potential of students to benefit fully from schooling as a result of having a positive health status [1-7]. The approach to health education at schools has changed in the last 20 years and is continuing to evolve. Twenty years ago, school health promotion was introduced in Europe as a new framework to assist schools in addressing health issues [8]. An integrated school approach with long-term implementation of health promotion programmes seemed to be more effective than short-term classroom-based programmes [7-10]. Yet the inclusion of extensive health promotion in school policies remains a challenge because education, and not health, is the core business of schools [11]. In recent years, many schools worldwide have implemented programmes to promote a healthy lifestyle and to counteract the increasingly unhealthy behaviour of school-age children, but evidence for the effectiveness of these interventions is scarce [12-15].

While there is consensus that an integrated school approach is the best way to promote health among secondary school children, little is known about the effect of combining different programmes to maximize benefit. An integrated school health promotion programme ('a healthy school') was started in 2007 at a single, public, secondary school in the region of Utrecht, the Netherlands. This programme covers a number of lifestyle aspects (physical activity, smoking, alcohol, nutrition, Internet behaviour, bullying and sexual behaviour) and replaces previous health education programmes. Research has shown that a broad strategy including several programmes for different lifestyles has a greater effect than a single programme focusing on one lifestyle at a time [16,17] Different teaching modules were developed for each class with the help of experts and in 2008 these were implemented in the teaching programme of the school. Parents were involved and a network of healthcare organizations was set up to facilitate appropriate treatment when problems were detected.

This article focuses on one health issue, namely, Internet use and gaming. Time spent on Internet and games is continuing to increase and there is concern about potential adverse effects on professional and academic life, social factors [18], alcohol use [19,20] and weight [21-23]. Heavy Internet and game use is called compulsive use and when compulsiveness is severe, it is called addictive. As early as in 1983, it was claimed video game use could become an addiction like any other behavioural addiction [24] and the same was argued for excessive Internet use several years later [25]. Internet (and game) addiction features the core components of addiction: salience, mood modification, tolerance, withdrawal, conflict and relapse [26,27]. It is difficult to diagnose Internet addiction because to date there are no accepted criteria for Internet or game addiction listed in the DSM IV [28]. The prevalence rates of addiction differ by country, probably due to different diagnostic tools [18].

There is little evidence that programmes to prevent addiction to Internet use and gaming are effective. One study stated that self-chosen coping strategies, such as replacing Internet use with real life activities, hiding electric cables, fines, etc, were ineffective and only helped participants to pass time until they next logged in [29]. It is important to promote awareness of Internet abuse or addiction and programmes should be implemented at schools/universities to decrease the potential dangers of excessive Internet use [29]. A study of the effects of a school-based online game addiction prevention programme for secondary school children showed that while the programme increased the school children's knowledge about online gaming and online game addiction, it did not bring about a significant change in self-control of online gaming [30].

The aim of this study was to investigate the heaviness/compulsiveness of Internet and game use among secondary school children and the preliminary effect of an Internet/game prevention programme. We also investigated whether Internet use and gaming are associated with other lifestyle factors, such as alcohol use and physical activity and with other factors that could influence this behaviour (BMI, psychosocial well-being and happiness at school and home). This project must be seen as a pilot and is the first step in a case-control study.

## Methods

In September 2007, a total of 1057 secondary school children (age range: 11-18 years) participated in the study and completed a 165-item anonymous paper-and-pencil questionnaire that covered health issues, personal and demographic characteristics and own beliefs. In total, 9.8% of the students did not take part in completing the questionnaire because they were absent from school that day. A year after the implementation of the prevention programme, the school children completed the same questionnaire to evaluate changes in behaviour. This second questionnaire was Internet based. A total of 475 children completed both questionnaires, 367 of them being first-, second-, or third-grade students who were taught about media behaviour. A lot of children who completed the first questionnaire did not complete the second questionnaire because they had left school after graduation. The data of these children were analysed.

The intervention used in this study was implemented by the school in 2007 as an improvement of the already existing health education. For the intervention and the questionnaire to be completed by the school children, no ethical approval was needed. Parents of all children declared consent for their children to participate in the health education project.

The questions used were derived from validated questionnaires. In this study, Internet use was defined as use of Internet for non-school related purposes. Game use was defined as online gaming or making use of X-Box, PlayStation etc. The Compulsive Internet Use Scale (CIUS), which is composed of 12 questions with a 5-point Likert scale, was used to evaluate Internet use [31]. An adapted (non-validated) version, the Compulsive Game Use Scale (CGUS) was used to evaluate game use. Game use did not include games with monetary awards or gambling. For both scales, the higher the score, the more compulsive the behaviour; pathological use was defined as an average score of >3. Students who did not use internet or gaming were included in these scales, giving them the lowest score possible.

Psychosocial problems were measured with the Strengths and Difficulties Questionnaire (SDQ) [32]. This is a scale composed of 5 subscales (emotional symptoms, hyperactivity, peer problems, conduct problems and prosocial behaviour); each composed of 5 questions scored 0, 1 or 2. The total SDQ score is the sum of the scores on the first 4 subscales (maximum score 40 points), with a higher score indicating more psychosocial problems. We used the total score of the self-reported SDQ and scores of 0-15 were classified normal, 16-19 borderline, and 20-40 abnormal [32].

A physical activity score was computed based on two questions about how often the students performed regular and intensive physical exercise for at least 30 minutes. A drinking score was computed, based on the average number of glasses of alcohol drank per week. Body Mass Index (BMI) was calculated as weight in kilograms divided by height in meters squared (self-scored). Family socioeconomic status was measured with the Family Affluence Scale (FAS), a 4-item measure of family wealth, developed in the WHO Health Behaviour in School-aged Children Study [33]. All these scores are based on self-report.

### Intervention

In September 2007, a health education programme for school children in the first three grades was started, during which the children received education on health issues for 2 hours a week. In the Netherlands, a secondary school can offer four levels of education: Lower General Secondary Education (4 years), Higher General Secondary Education (5 years) and pre-university education (6 years), which consists of two levels, namely, with or without Greek or Latin as a final examination subject. Children in all first, second and third grade classes, except for the third grade class of pre-university education with Greek or Latin, were taught about media behaviour by their own teachers. The intervention developed was based on existing literature about media literacy [34]. School children learnt to cope with the possibilities, deceptions and dangers of internet. The lessons covered several aspects of Internet use, such as digital communication, online bullying, online imago, online sexuality, distorted beauty ideal and Internet advertisements. The teachers were trained and assisted by experts from local health agencies (GGDs) and from institutes specialized in diagnosing and treating addiction (Centrum Maliebaan). The lesson content was adapted to the class and educational level of the children.

### Data analysis

Data were analysed for the sample as a whole and for the different educational groups. In addition, second, third and fourth grade school children from 2007 were compared with second, third and fourth grade schoolchildren from 2008 respectively, to check for the effects of maturation. As mentioned above, pathological use was defined as a mean CIUS or CGUS score >3. Statistical analyses were carried out using SPSS for Windows, version 15.0. Chi-square tests and T-tests (independent and paired) were used for categorical and continuous variables, respectively. Cochrane Q was used to evaluate differences over time for categorical variables. ANOVA was used to detect covariates and afterwards univariate ANCOVA was used to correct for these covariates.

## Results

The baseline characteristics of the children who completed both questionnaires are presented in Table 1. Most children were in first or second grade, followed pre-university education, were of Dutch ethnicity and came from above-average income families. Most children were aged between 11 and 14 years old. 4% was of the age of 15-16 years and these were mostly children who doubled a class and therefore were still in third grade. Children with the highest (top 20%) scores for Internet use and gaming (referred to hereafter as "heavy users") were compared with children with the lowest (bottom 20%) scores on the relevant scales (referred to hereafter as "non-heavy users") (see Table 2). As can be expected, there is an overlap in population groups of heavy Internet users and heavy game users.

**Table 1:** Baseline characteristics

Variables	N (%) Total = 367
**Gender**	
Male	144 (40.8)
Female	209 (59.2)
**Age in years (11-16)**	
11-12	122 (33.3)
13-14	230 (62.7)
15-16	15 (4.0)
**Year of school**	
1	116 (31.5)
2	176 (47.8)
3	76 (20.7)
**Level of secondary education**	
Lower General (VMBO)	54 (14.7)
Higher General (HAVO)	103 (28.1)
Pre-university (VWO)	110 (30.0)
Pre-university with Greek/Latin (Gymnasium)	100 (27.2)
**Ethnicity**	
Dutch	314 (85.3)
European	25 (6.8)
Other	29 (7.9)
**FAS^1^**	
Low (0, 1 or 2)	1 (0.3)
Medium (3,4 or 5)	84 (23.5)
High (6-7)	272 (76.2)

^1^Family Affluence Scale

**Table 2:** Comparison of <20% and > 20% CIUS scores and <20% and > 20% CGUS scores of M1

	All schoolchildren Mean (SD) N = 367	< 20% CIUS Mean (SD) Nmax = 71	> 20% CIUS Mean (SD) Nmax = 72	< 20% CGUS Mean (SD) Nmax = 70	> 20% CGUS Mean (SD) Nmax = 49
Hours of internet use/day	1.46 (2.00)	0.98 (1.18)	2.45** (3.26)	2.09 (2.37)	2.20 (3.75)
Hours of game use/week	2.99 (5.00)	2.65 (3.49)	3.24 (6.07)	1.10 (1.21)	6.07** (7.37)
CIUS score^1^	1.64 (0.50)	-	-	1.53 (0.50)	2.03** (0.60)
CGUS score^2^	1.42 (0.44)	1.29 (0.30)	1.68** (0.72)	-	-
Drinking score	1.22 (2.97)	1.00 (3.22)	1.83 (3.00)	1.34 (3.07)	0.93 (1.46)
Physical activity score	5.69 (0.99)	5.82 (0.97)	5.48 (1.19)	5.83 (0.98)	5.65* (0.97)
SDQ score	9.54 (4.20)	7.80 (4.34)	11.26** (3.93)	9.36 (4.68)	10.88* (4.55)
BMI	18.29 (3.34)	18.14 (3.70)	18.84 (2.37)	18.22 (2.82)	17.93 (3.39)
Happiness at home	80.81 (20.58)	86.15 (18.23)	75.03* (22.30)	80.88 (21.50)	76.39* (25.79)
Happiness at school	73.23 (19.31)	77.61 (17.90)	64.79** (20.62)	75.99 (19.70)	66.55** (19.59)

^1^Compulsive Internet Use Scale * p ≤ 0.05 (after adjusting for covariates)

^2^Compulsive Game Use Scale ** p ≤ 0.01 (after adjusting for covariates)

Independent T-test and Univariate Analysis of Variance

### Heavy Internet Use groups

Heavy Internet users had more behavioural problems (a higher SDQ score). 13.8% of heavy users scored borderline vs 1.4% of non-heavy users. In both groups one child scored pathological. Heavy internet users were also less happy at home and at school, and had a higher CGUS score. Girls were more likely than boys to be heavy Internet users (24.3% of all girls vs 17.4% of all boys; p < 0.005) and in general scored higher on the CIUS (p < 0.05). Moreover, a relatively high proportion of children in the Lower General Secondary Education group were heavy Internet users (33.9% vs 7.1% non-heavy users). The FAS scores (socioecomomic status) were not significantly different between heavy and non-heavy Internet users. One-way ANOVA identified sex, age category and educational level as covariates. Results were still significant after correction for these covariates.

### Heavy Game Use groups

Heavy game users had more behavioural problems (a higher SDQ score). 14.3% of heavy game users scored borderline vs 4.3% of non-heavy users. In both groups one child scored pathological. Heavy game users were also less happy at school and had a higher CIUS score than non-heavy game users. In contrast to heavy Internet use, boys were more likely than girls to be heavy game users (29.5% vs 7.7%; p < 0.001). Boys also had a higher CGUS score (p < 0.001) and spent more time playing computer games (p < 0.001). One-way ANOVA identified sex to be the only covariate. After correction for sex, the results remained significant, and those for physical activity, SDQ score and happiness at home became significant (p < 0.05).

### Changes after the intervention

Table 3 shows the Internet and game use of the school children before and after the intervention (paired T-test). There was a significant increase in the number of hours/day spent on Internet and in the number of children who could be considered pathological Internet users. While there was a significant decrease in the number of children who played computer games, the CGUS scores of those who did play increased significantly. The school children did not indicate that the intervention influenced their intention to change Internet and game use.

**Table 3:** Internet and game use before (M1) and a year after the start of the prevention program(M2)

Variables	M1^3 ^N (%) Total = 367	Mean (SD)	M2^4 ^N (%) Total = 367	Mean (SD)
Internet use				
Yes	350 (95.6)		359 (97.6)	
No	16 (4.4)		9 (2.4)	
Hours per day		1.44 (2.00)		2.22** (1.88)
CIUS score^1^		1.64 (0.50)		1.67 (0.62)
Pathologic No	364 (98.9)		356 (96.7)	
Pathologic Yes	4 (1.1)		12 (3.3)*	
Game use				
Yes	264 (72.5)		236 (64.1)	
No	100 (27.5)		132 (35.9)**	
Hours per week		3.24 (5.11)		4.3 (7.44)
CGUS score^2^		1.42 (0.45)		1.47* (0.44)
Pathologic No	364 (98.9)		362 (98.4)	
Yes	4 (1.1)		6 (1.6)	

^1^Compulsive Internet Use Scale * p ≤ 0.05

^2^Compulsive Game Use Scale ** p ≤ 0.01

^3^First measurement

^4^Second measurement

Paired Samples T-Test.

The same analyses were also performed for children following the different levels of education. To this end, we combined the data for all children (grades one, two and three) following the different types of education and compared changes over time (paired T-test) (Table 4). Hours of Internet use increased in the three highest educational levels.

**Table 4:** Differences split out for different education levels

Variables	M1^3 ^Mean (SD) Total = 367	M2^4 ^Mean (SD) Total = 367
**Lower General Secondary Education Nmax = 54^**		
Internet hours/day	2.85 (3.80)	2.94 (1.84)
CIUS score^1^	1.90 (0.57)	1.88 (0.74)
Game hours/week	2.21 (2.38)	5.90 (11.43)
CGUS score^2^	1.46 (0.56)	1.56 (0.66)
		
**Higher General Secondary Education** **Nmax = 103**		
Internet hours/day	1.55 (1.88)	2.63** (2.22)
CIUS score	1.62 (0.51)	1.69 (0.60)
Game hours per week	4.04 (5.89)	4.63 (8.76)
CGUS score	1.43 (0.43)	1.45 (0.42)
		
**Pre-university education** **Nmax = 110**		
Internet hours/day	1.07 (0.86)	2.04** (1.88)
CIUS score	1.59 (0.46)	1.62 (0.56)
Game hours/week	3.65 (5.82)	4.27 (5.60)
CGUS score	1.39 (0.46)	1.47* (0.38)
		
**Pre-university education + greek/latin Nmax = 100**		
Internet hours/day	1.05 (1.31)	1.66** (1.25)
CIUS score	1.58 (0.44)	1.61 (0.61)
Game hours/week	2.59 (4.58)	3.14 (3.89)
CGUS score	1.41 (0.37)	1.44 (3.78)

^1^Compulsive Internet Use Scale * p ≤ 0.05

^2^Compulsive Game Use Scale ** p ≤ 0.01

^ The sample of game hours/week is smaller because a lot of students do not use games.

Paired samples T-test

The same analyses were performed separately for second, third and fourth grade school children to overcome the effects of maturation: second graders of 2007 (who had not yet followed the education programme) were compared with second graders of 2008 (who had followed the education programme), etc. (independent T-test) (Table 5). After the intervention, in 2008, Internet use in second year school children increased (both hours and CIUS score); children of the third year had a higher CIUS score, and children of the fourth year spent more hours on Internet use and had a higher CGUS score.

**Table 5:** Differences split out for different grades

Variables	M1^3 ^Mean (SD) Total = 367	M2^4 ^Mean (SD) Total = 367
**Second year**	Nmax = 176	Nmax = 128
Internet hours/day	1.42 (1.71)	2.12** (1.81)
CIUS score^1^	1.54 (0.42)	1.72** (0.60)
Game hours/week	2.80 (3.32)	3.18 (5.60)
CGUS score^2^	1.39 (0.38)	1.46 (0.40)
		
**Third year**	Nmax = 76	Nmax = 164
Internet hours/day	2.15 (3.19)	2.07 (1.86)
CIUS score	1.85 (0.60)	1.52** (0.57)
Game hours per week	2.77 (5.06)	4.15 (6.68)
CGUS score	1.43 (0.51)	1.14 (0.38)
		
**Fourth year^3^**	Nmax = 54	Nmax = 74
Internet hours/day	1.79 (1.55)	2.74(*) (2.42)
CIUS score	1.78 (0.64)	1.93 (0.67)
Game hours per week	2.53 (3.93)	4.37 (9.99)
CGUS score	1.40 (0.48)	1.61(*) (0.61)

^1^Compulsive Internet Use Scale * p ≤ 0.05

^2^Compulsive Game Use Scale ** p ≤ 0.01

^3 ^The group fourth year students also consists of students who were in their fourth year in 2007. They did not take part in the prevention program and therefore are not part of the population sample (N = 367) we used for all other analyses.

(Independent T-test and Univariate Analysis of Variance).

(*) = lost significance after correction for covariates

One-way ANOVA did not identify covariates in the group of second-grade children but found sex and educational level to be covariates in both third- and fourth-grade children. When we corrected for these covariates, differences in Internet hours/day and CGUS between the fourth-grade children were no longer significant.

## Discussion

Only 1.1% of the school children could be considered pathological Internet or game users at baseline. In general, heavy Internet users spent significantly more hours on Internet, showed more heavy game use, had more psychosocial problems and were more often unhappy at school and at home than non-heavy Internet users. Likewise, heavy game users spent more hours on gaming, showed more heavy Internet use, had more psychosocial problems and were less physically active than non-heavy game users. Heavy Internet and game use was not related to alcohol use or BMI. Thus heavy Internet and game use is not an isolated problem but is accompanied by other health issues. This means that school health education programmes should focus on more than one health issue at a time, as did the programme we tested.

A year after the education programme had started, we found the number of hours spent daily on Internet had increased, as had the number of pathological Internet users. While the number of game users had decreased, the heaviness of game use had slightly increased.

The differences between the various levels of education were minor. Second grade school children in 2008 spent more time on Internet (hours/day), were more heavy Internet users and were more often pathological Internet users compared with second grade school children in 2007. In contrast, there was a marked decrease in heavy Internet use and a non-significant decrease in CGUS scores among third grade school children in 2008 compared with 2007. Heavy Internet (or game) use is associated with more hours spent on game (or Internet) use, more heavy game use (or Internet) and more problematic behaviour.

School children following a lower general secondary education were more likely to be heavy Internet users than school children following other educational levels. This relationship between heavy Internet use and education level has been reported earlier for 13- to 15-year-old school children [35]. We did not find heavy Internet or game use to be associated with alcohol consumption among the school children, which is in contrast with the results of Ko et al. (using other questionnaires) [19,20]. Moreover, in contrast with other investigators [36], we found no significant relationship between heavy Internet use and physical activity; however, we did find physical activity to be negatively associated with more heavy game use, but we did not find literature for comparison. Heavy Internet and game users reported more psychosocial problems, as reported by Cao and Su [37] for Internet addicts. We found no relationship between heavy Internet/game use and BMI, in contrary to many studies that did [21-23].

The increased time spent on Internet (hours/day) by the school children in 2008 compared with the same school children in 2007 is consistent with national trends of increased frequency and duration of Internet use with increasing age among secondary school children [38,39]. We also found that second grade children spent more time on Internet in 2008 than in 2007 which is consistent with the increased use of Internet reported among school children in recent years [32]. However, we did not find an increase in Internet use among third or fourth grade school children. The number of pathological Internet users increased over time, which is contrary to a previous Dutch report which reported a decrease (4.2% in 2006, 3.6% in 2007, and 3.2% in 2008) [38]. The proportion of children who played computer games (64%) was comparable to that reported in previous studies [38]. We found a lower proportion of pathological game users than reported previously (1.6% vs 3.2%) but results are not quite comparable because different measurement scales were used [38].

Some limitations of this study should be mentioned. The school at which the intervention took place is not an average Dutch secondary school. In this school, most school children are of Dutch ethnicity, come from a high socioeconomic background and follow higher levels of education. Therefore, the amount of problematic behaviour is almost certainly less than the national average and implementation of the education programme was probably easier than in an average secondary school. It can be hypothesized that effects of the prevention program might be greater at schools with more problematic behaviour. Unfortunately, we did not use a control school at this stage of the study and thus changes in Internet and game behaviour over time must be interpreted with caution. It is not clear in what way the education programme contributed to the changes in Internet and game use and how use would have changed over time without the education programme, as a result of government initiatives, parenting or television programmes on the subject. Moreover, the school children might have become more aware of their behaviour when they completed the questionnaire for the first time, which led to changes when completing it the second time. The questionnaire measuring game use and the cut-off score for heavy game use is based on the validated questionnaire measuring internet use, but is not yet validated itself. Therefore, results of this study regarding game use and its generalizability have to be interpreted carefully. It is unlikely that the computer based follow-up questionnaire (in comparison to the paper-and-pencil questionnaire) caused measurement bias as several studies have shown results between these different methods are comparable [40]. Finally, all computed scores of behavioral factors were based on self report and could therefore have resulted in some reporting bias. But even taking these limitations into account, we can draw some interesting conclusions from our data.

As mentioned before, heavy Internet and game use was associated with more psychosocial problems, less physical activity, and greater unhappiness. The question is which comes first - do heavy Internet and game users have more psychosocial problems because of their lack of interactions with family and friends in real life, or do school children lacking social interactions choose Internet and game use as an easier way to communicate with other people. Other studies have found that "chat" users are socially fearful and may use the Internet as a form of low-risk social approach and an opportunity to rehearse social behaviour [35]. The decrease in physical activity suggests that time spent on gaming goes at the cost of time spent on physical activity. However, other studies have reported that Internet and game use does not increase leisure time but replaces time spent on other leisure activities [41]. The observation that heavy users were more often unhappy at school and at home is important and highlights the importance of detecting heavy Internet and game use and of providing support, treatment and preventive measures at school.

The fairly large increase in time spent on Internet probably reflects the combined influence of an increase in use over the last years and an increase in use with increasing age. Despite the increased time spent on Internet, CIUS scores did not change, which suggests that spending more time on the Internet does not lead to more heavy Internet use. It may also be possible that the CIUS is not sensitive enough to detect such changes or that hours of Internet use and heaviness of internet use are not linearly associated. The decrease in the number of game users with time was noticeable and might be due to the education programme but more evaluations over time are needed to see whether this trend continues. The fact that Internet use increased among second grade children but not among third or fourth grade children raises the question whether second grade school children are less amenable to the influence of the educational programme.

## Conclusions

In conclusion, we found some evidence that a health education programme encompassing different health issues and peer education has a beneficial effect on health behaviour in secondary school children. A larger controlled, longitudinal study is needed to further evaluate the effects of this school health promotion approach.

## Abbreviations

BMI: Body Mass Index; CGUS: Compulsive Game Use Scale; CIUS: Compulsive Internet Use Scale; FAS: Family Affluence Scale; SDQ: Strengths and Difficulties Questionnaire.

## Competing interests

The authors declare that they have no competing interests.

## Authors' contributions

All authors read and approved the final manuscript. MB: Data analyses and description of main results; GHW: critical screening of the paper and feedback; AJPS: critical screening of the paper and feedback;

JRJL: Coordination of data analyses and description of the results.

## Pre-publication history

The pre-publication history for this paper can be accessed here:

http://www.biomedcentral.com/1471-2458/10/544/prepub
